# Supplementary material for: Advanced computational approaches for predicting sunflower yield: Insights from ANN, ANFIS, and GEP in normal and salinity stress environments
Source: PLoS One. 2025 Feb 24;20(2):e0319331. doi: 10.1371/journal.pone.0319331 (PMC11849905; doi:10.1371/journal.pone.0319331)
Supplement: Attachment — Submitted filename: Response to reviewer comments.docx [file pone.0319331.s001.docx]

January 3, 2025

### Dear Editor-in-Chief,

I would appreciate it if you look into our revised manuscript entitled “Advanced Computational Approaches for Predicting Sunflower Yield: Insights from ANN, ANFIS, and GEP in Normal and Salinity Stress Environments”. As far as the referee’s propositions are concerned, all modifications have been applied and highlighted for your consideration. The manuscript was critically revised by an English-speaking native for spelling and grammar. We the thank referees for their valuable recommendations and hope that they will be satisfied with the revised manuscript.

Sincerely yours

Corresponding author

**Response to reviewer comments:**

**The answer to the comments of the first referee**

It should be noted that the suggestions of the first respected referee are highlighted in green in the text.

**Answer to first question**

Rationale for Selecting Salinity Levels (2 and 8 dS/m)

Practical agricultural considerations and the physiological responses of sunflower plants to salinity stress guided the selection of salinity levels at 2 and 8 dS/m. The following key points explain the rationale behind these choices:

1. Baseline Control (2 dS/m): A salinity level of 2 dS/m was chosen to represent the normal irrigation water salinity in the study region (Nazlou, Iran). This level serves as the control, reflecting standard growth conditions. This baseline aligns with typical irrigation practices in the experimental area, enhancing the study's relevance to local farming conditions.

2. Stress Level (8 dS/m): The 8 dS/m salinity level was selected to simulate moderate salinity stress commonly encountered in arid and semi-arid regions, where sunflower cultivation is prevalent. According to Maas and Hoffman (1977), sunflower is categorized as a relatively sensitive crop, with yield reductions starting at salinity levels above 4 dS/m. The 8 dS/m level was chosen to induce measurable stress responses without causing complete plant failure. This level is consistent with findings by Katerji et al. (2000), who observed significant physiological and yield responses in sunflower at salinity levels ranging from 6 to 10 dS/m. Thus, 8 dS/m represent a practical threshold for moderate stress, enabling meaningful comparisons of morphological traits and yield between normal and stressed conditions.

3. Relevance to local agricultural practices: The selected salinity levels aim to impose stress that does not entirely incapacitate the plants but allows for measurable changes in performance. Farmers in the region often face irrigation water salinity levels between 2 and 8 dS/m due to factors such as soil salinity and limited access to freshwater resources. Therefore, these levels enhance the study's relevance to real-world scenarios and support the development of predictive models that can guide irrigation strategies and crop management under varying salinity conditions.

By selecting these salinity levels, the study ensures both scientific validity and practical applicability, facilitating the development of tools to predict and manage sunflower production under diverse environmental conditions.

**Answer to second question:**

**Justification for Model Selection: Why ANN, ANFIS, and GEP?**

The selection of Artificial Neural Networks (ANN), Adaptive Neuro-Fuzzy Inference Systems (ANFIS), and Gene Expression Programming (GEP) was underpinned by their demonstrated ability to model complex and nonlinear relationships in agricultural and environmental datasets, particularly under abiotic stresses like salinity. A detailed explanation for their inclusion is provided below:

1. **Artificial Neural Networks (ANN):**

- **Flexible Nonlinear Modeling:** ANNs are well-suited for capturing complex nonlinear relationships between input variables (morphological features) and outputs (grain yield). Their ability to learn from data without rigid assumptions makes them powerful for yield prediction.
- **Proven Success in Agriculture:** Previous research has shown that ANNs can outperform traditional linear regression models in prediction accuracy when dealing with agricultural data and yield estimation.
- **Adaptability:** ANNs can be easily adjusted in terms of architecture (number of layers and neurons), learning algorithms, and activation functions, allowing for optimal performance on specific datasets.

2. **Adaptive Neuro-Fuzzy Inference Systems (ANFIS):** ANFIS combines the learning capabilities of ANNs with the interpretability of fuzzy logic. This made it a suitable choice due to its ability to model uncertain and imprecise data, a common challenge in agricultural studies. ANFIS can emulate human-like reasoning and provide rule-based outputs that are easily interpretable, beneficial for translating findings into practical agricultural practices.
3. **Gene Expression Programming (GEP):** GEP was included due to its strength in symbolic regression and its ability to evolve models that explicitly explain the relationships between variables in mathematical form. This feature is crucial for generating predictive models that not only forecast outcomes but also provide insights into the underlying mechanisms of stress responses.

While ANN, ANFIS, and GEP were selected for their respective strengths, other modeling methods could have been considered, such as:

- **Support Vector Machines (SVM)**
- **Random Forest (RF)**
- **Gradient Boosting Machines (GBM) and XGBoost**
- **Deep Learning (e.g., Convolutional Neural Networks - CNN, Recurrent Neural Networks - RNN):** Deep learning models could be considered for handling large and complex datasets, especially when spatial or temporal dynamics are important.

**Justification for not including other models:** While models like SVM, RF, and GBM are powerful, the primary motivation for selecting ANN, ANFIS, and GEP was their combined ability to handle nonlinearity, uncertainty, and interpretability. This combination is critical in agricultural studies, where accurate prediction and understanding of underlying processes are vital. The selected models effectively meet the study's goals of predicting outcomes and elucidating plant responses to salinity stress.

**Answer to third question**

Data Preparation and Preprocessing

A dataset encompassing 104 sunflower recombinant inbred lines was evaluated under two normal and salinity stress conditions. After data collection, missing values within the dataset were identified. This process involved meticulously examining each trait to identify gaps in the data inputs. Subsequently, lines with a high proportion of missing values and data points identified as outliers and deemed measurement errors were removed from the dataset. This action was taken to preserve the model's integrity under normal and salinity stress conditions. Following this step, the mean values for all data points were calculated. Next, the dataset of mean values was divided into training and testing subsets using a 75:25 ratio, as previously described in the text. This division ensures that the models are trained solely on one dataset while being validated on an independent subset. This approach allows for an unbiased evaluation of model performance and enhances the accuracy of the generated predictions.

4-**The fourth question**

Model training details: Can you elaborate on the training process for each model?

**1. Artificial Neural Networks (ANN)**

- Training Process:
- Initialization: The ANN model begins with the establishment of its architecture, which includes an input layer corresponding to the 10 morphological traits, one or more hidden layers, and an output layer designed to predict grain yield.
- Data Preparation: The training dataset, consisting samples obtained from a 75:25 split, is utilized to fit the model.
- Hyperparameter Tuning
- Number of Hidden Layers and Neurons: An iterative trial-and-error methodology was employed to test various configurations of hidden layers, specifically ranging from 1 to 10 neurons per layer. Model performance was evaluated using mean squared error (MSE) until optimal configurations that minimized prediction error were identified.
- Activation Functions: Different activation functions—including sigmoid, hyperbolic tangent, and linear—were explored for both hidden and output layers to effectively capture non-linear relationships within the data.
- Training Algorithm: The Levenberg-Marquardt backpropagation algorithm was used for its efficiency in model training. This second-order optimization technique helps facilitate rapid convergence to a solution with minimal error.
- Training Epochs: The training procedure was designed to continue until the MSE fell below a threshold of 0.001 or until a maximum of 1,000 epochs was reached, establishing a clear convergence criterion.

**2. Adaptive Neuro-Fuzzy Inference System (ANFIS)**

- Training Process:
- Model Setup: ANFIS integrates fuzzy logic with neural networks, utilizing a Sugeno-type rule-based system. The initial setup entails selecting input parameters and defining relevant membership functions.
- Clustering: A subtractive clustering method was employed to ascertain the number of fuzzy rules and corresponding membership functions. Each input data point served as a potential cluster center, contributing to the optimization of model complexity.
- Hyperparameter Tuning:
- Radius of Influence (RADII): The RADII parameter is integral to the subtractive clustering approach as it regulates the granularity of fuzzy partitioning. Through experimentation, optimal RADII values of 0.32 and 0.26 were identified for normal and stressed conditions, respectively.
- Training Duration: Consistent with the ANN approach, training continued until the MSE fell below the threshold of 0.001, or until a maximum of 1,000 epochs was reached, providing uniformity in stopping criteria.

**3. Gene Expression Programming (GEP)**

- Training Process:
- Genetic Algorithm Structure: GEP merges principles from genetic algorithms and genetic programming, initiating with a population of randomly generated chromosomes that represent potential solutions.
- Fitness Evaluation: The fitness of each chromosome is assessed based on its accuracy in predicting sunflower grain yield, typically measured using metrics such as MSE or RMSE.
- Hyperparameter Tuning:
- Population Size: The population size was fixed at 30 chromosomes, influencing genetic diversity and the potential for uncovering optimal solutions.
- Head Length and Gene Count: Default configurations were set with a head length of \(h =7\) and three genes per chromosome, based on the parameters established within the GeneXpro tools applied for GEP.
- Genetic Operators: Throughout various generations, a range of genetic operations—including mutation, crossover, and selection—were implemented. Specific operator settings were closely monitored to ensure a balanced trade-off between exploration (diversity) and exploitation (convergence toward optimal solutions).

**Performance Evaluation and Final Model Selection**

Cross-validation: Post-training, models were evaluated against the testing dataset to ascertain their generalizability to unseen data. Metrics such as (R^2^), RMSE, and MAE were utilized to gauge the accuracy of predictions.

Model Comparison: The derived performance indicators facilitated a comparative analysis among the ANN, ANFIS, and GEP models, allowing for the selection of the best-performing model based on criteria of accuracy and interpretability.

In summary, each model underwent meticulous training with a focus on hyperparameter tuning through diverse methodologies: trial-and-error for the ANN, parameter adjustments through clustering in ANFIS, and genetic operations in GEP. The overarching objective was to enhance model performance while ensuring robustness and generalizability in predicting sunflower grain yield across varying environmental conditions.

**Answer to question five**

**5. Validation Methods: What methods were used to validate the models? Were cross-validation techniques employed?**

Validation of ANN, ANFIS, and GEP Models:

The models (ANN, ANFIS, and GEP) were validated using a split-sample approach to assess predictive accuracy, generalization, and robustness. Below are the validation details:

1. Dataset Division:

- Training Set: 75% (216 samples) used for model training.
- Testing Set: 25% (72 samples) reserved for independent evaluation to ensure generalization and avoid data leakage during training.
- This division effectively mirrored real-world conditions, where models must predict outcomes for unseen data.

2. Performance Metrics

The models were evaluated using three key metrics:

- Coefficient of Determination (R²): This indicates how well the model predictions align with observed values, with values closer to 1 reflecting better model performance.
- Root Mean Squared Error (RMSE): Measures the magnitude of prediction errors, penalizing larger errors more heavily.
- Mean Absolute Error (MAE): This provides a more balanced evaluation by averaging absolute prediction errors, treating both small and large errors equally.

3. Cross-Validation Techniques:

Although cross-validation (e.g., k-fold) was not explicitly used, the following steps ensured robust model evaluation:

- Train-Test Split: Mimics cross-validation by using a separate testing set to evaluate model generalizability.
- Repeated Trials: Hyperparameters were optimized through multiple iterations of training, ensuring model consistency across different configurations.
- Sensitivity Analysis (GEP Model): Identified key input features (e.g., leaf length, head diameter) that significantly influenced model predictions, indirectly validating the relevance of selected features.

4. Graphical Validation:

- Scatter Plots (Predicted vs. Observed): Visualized how closely model predictions matched actual yield values, with regression lines and R² values showing prediction accuracy.
- Taylor Diagrams: Combined correlation, RMSE, and standard deviation into a single graph for a comprehensive performance assessment.
- Violin Plots: Compared distributions of predicted and observed values, highlighting the models' ability to capture data variability.

5. Key Findings from Validation:

- GEP Model:

Best performance with R² = 0.803 (normal conditions) and R² = 0.743 (salinity stress), showing the highest accuracy, lowest RMSE, and MAE across conditions. Its explicit mathematical equations allowed for deeper interpretation and validation.

- ANFIS Model:

Achieved intermediate accuracy with R² = 0.758 (normal) and R² = 0.668 (salinity stress). This model handled nonlinear relationships well through fuzzy logic, but its performance was slightly lower than the GEP model.

- ANN Model:

Lower performance with R² = 0.716 (normal) and R² = 0.634 (salinity stress), possibly due to its black-box nature, which made it more prone to overfitting and less interpretable than the other models.

The GEP model outperformed both ANFIS and ANN models in terms of predictive accuracy and robustness under both normal and salinity stress conditions. The validation process using multiple metrics and graphical methods confirmed the reliability of the models, with GEP offering the most effective solution for sunflower yield prediction under varying environmental conditions.

**Answer to six question**

In this study, sensitivity analysis was not conducted, and the parameters (LL, HD, HSW, and HDW) were identified as influential characteristics affecting sunflower seed yield and modeling using the GEP model under salt stress conditions, rather than as components related to sensitivity analysis. The following sections will elaborate on the role and significance of these parameters in impacting sunflower performance under salinity stress.

Leaf length is crucial due to its direct relationship with the plant's potential photosynthetic capacity. Longer leaves are capable of absorbing more sunlight, which is vital for the process of photosynthesis (Ren et al., 2019; Niinemets, 2010). Under salinity stress conditions, plants may experience reduced water availability and increased osmotic pressure, leading to physiological responses that limit growth. Therefore, maintaining effective leaf length is essential for maximizing photosynthesis and the overall efficiency of the plant (Ahmad et al., 2014; Farooq et al., 2019).

Salinity-induced stress can lead to reduced turgor pressure in leaves, resulting in wilting or leaf curling. A significant decrease in leaf length may indicate the presence of stress, which directly impacts the overall health of the plant and seed yield.

The head diameter of sunflowers, as a vital trait associated with the reproductive organs of flowers, significantly influences seed growth and development of sunflowers. The size of the head diameter impacts the number of flowers and seeds per capitulum, directly affecting seed yield per plant (Hladni et al., 2014). Consequently, a substantial head diameter under salinity stress conditions may indicate the plant's resilience in maintaining reproductive success, even when faced with adverse growth conditions. Rai et al. (2006) reported a strong positive correlation between seed yield, the number of filled seeds per plant, seed set percentage, head diameter, and the harvest index. They emphasized that selection for traits such as the number of filled seeds per capitulum, average capitulum diameter, higher test weight, average plant height with greater leaf area, and total dry matter per plant could significantly enhance seed yield in sunflowers. In a study addressing seed retention and filling challenges in sunflowers, Ram and Davari (2011) noted that increasing stem diameter and head diameter to a certain extent could lead to a higher number of filled seeds per capitulum. Additionally, Hassan et al. (2013) demonstrated that selection based on key traits such as head diameter, seed weight, plant height, and base stem diameter could more effectively contribute to improving seed yield in various sunflower genotypes.

These findings underscore the importance of morphological and physiological factors as key criteria in genetic improvement programs for sunflowers under salinity stress. The integration of these traits into breeding strategies could lead to enhanced performance and yield stability in challenging environmental conditions.

HSW (Hundred Seed Weight) is a crucial component of yield and serves as a significant indicator of seed quality and nutritional value. Any changes in this metric can lead to variations in sunflower yield (Singh et al., 2018). Under salinity stress conditions, plants often allocate more resources to seed development, even if the overall yield (number of seeds) is adversely affected. A higher HSW may suggest that the plant continues to produce viable seeds despite stress, reflecting its adaptability.

Excessive salinity tolerance ultimately results in yield loss, representing one of the most quantifiable impacts in agriculture from a breeding perspective. Research conducted by Nahar and Hasanuzzaman (2009) demonstrated that salinity negatively affects yield and its components, including the number of pods per plant, seeds per pod, and seed weight, leading to significant reductions.

Moreover, variations in thousand-seed weight can be attributed to genetic factors among different genotypes, as well as to the processes of seed formation and development, which are influenced positively or negatively by soil and environmental conditions (Radić et al., 2013). HSW reflects the quality and viability of sunflower seeds and is a critical indicator for understanding how salinity stress impacts overall yield potential.

Head dry weight (HDW) reflects the total biomass allocated to the head region of a plant, which is essential for seed formation. Plants that can maintain head dry weight under saline conditions likely exhibit enhanced physiological functions, such as efficient nutrient uptake and water management, which are critical for coping with stress.

The combination of these traits is particularly important due to their interconnected roles in plant growth and reproductive success under salt stress. The identification of these traits as significant under stress conditions highlights their role in the adaptive strategies employed by sunflowers to balance growth and reproduction in challenging environments.

Specifically, traits such as leaf length, capitulum diameter, 1000-seed weight, and head dry weight are crucial under salinity stress, as they directly influence the physiological health, reproductive performance, and ultimately the yield potential of the plant. Understanding these factors can guide breeders and farmers in selecting and managing sunflower lines with enhanced salinity tolerance, thereby improving overall productivity in adverse conditions.

Hassan, S. F., Iqbal, M. S., Rabbani, G., Shabbir, G., Riaz, M., & Noorka, I. R. (2013). Correlation and path analysis for yield and yield components in sunflower (Helianthus annus. L). African Journal of Biotechnology, 12(16).

Ram, M., & Davari, M. R. (2011). Seed setting and filling problem in sunflower and its management–A. International Journal of Agronomy and Plant Production, 2(2), 33-56.

Hladni, N., Miklič, V., Jocić, S., Kraljević-Balalić, M., & Škorić, D. (2014). Mode of inheritance and combining ability for plant height and head diameter in sunflower (Helianthus annuus L.). Genetika, 46(1), 159-168.

Ravi, E., Bharathi, M., Reddy, A. V., & Ganesh, M. (2006). Character association and path analysis for seed yield in sunflower, Helianthus annuus L. Journal of Oilseeds Research, 23(1), 43.

Nishat, L. (2020). Diversity analysis of sunflower germplasms for yield and yield contributing characters (Doctoral Dissertation, Department of Agroforestry and Environmental Science, Sher-e-Bangla Agricultural University, Dhaka).

Nahar, K., & Hasanuzzaman, M. (2009). Germination, growth, nodulation and yield performance of three mungbean varieties under different levels of salinity stress. Green Farming, 2(12), 825-829.

Radić, V., Mrđa, J., Jocković, M., Čanak, P., Dimitrijević, A., & Jocić, S. (2013). Sunflower 1000-seed weight as affected by year and genotype. Ratarstvo i povrtarstvo/Field and Vegetable Crops Research, 50(1), 1-7.

Ren, T., Weraduwage, S. M., & Sharkey, T. D. (2019). Prospects for enhancing leaf photosynthetic capacity by manipulating mesophyll cell morphology. Journal of Experimental Botany, 70(4), 1153-1165.

Niinemets, Ü. (2010). A review of light interception in plant stands from leaf to canopy in different plant functional types and in species with varying shade tolerance. Ecological Research, 25, 693-714.

Farooq, M., Hussain, M., Ul-Allah, S., & Siddique, K. H. (2019). Physiological and agronomic approaches for improving water-use efficiency in crop plants. Agricultural Water Management, 219, 95-108.

Ahmad, R., Waraich, E. A., Ashraf, M. Y., Ahmad, S., & Aziz, T. (2014). Does nitrogen fertilization enhance drought tolerance in sunflower? A review. Journal of plant nutrition, 37(6), 942-963.

Singh, V. K., Sheoran, R. K., & Chander, S. (2018). Correlation analysis for seed yield and its component traits in sunflower. *Journal of Pharmacognosy and Phytochemistry*, *7*(3), 2299-2301.

**Answer to Seventh question**

**7. Comparison with Previous Studies: How do the results of this study compare with previous studies on sunflower yield prediction under salinity stress?**

Comparing the results of this study regarding the prediction of sunflower performance under salt stress with previous studies provides a deeper understanding of advancements in modeling approaches, the importance of specific traits, and the overall insights gained from field experiences.

Key Points of Comparison:

As previously discussed, the key traits identified in this study align with the findings from prior research. This study recognized leaf length (LL), head diameter (HD), a hundred seed weight (HSW), and head dry weight (HDW) as effective traits influencing sunflower performance. Previous studies have similarly highlighted the roles of these morphological and physiological traits in predicting sunflower yield under saline conditions. For instance, in our prior research conducted on a distinct set of 100 sunflower inbreeds, we identified leaf length as the most influential trait affecting seed yield in both normal and drought stress conditions when comparing impactful input variables in deep learning modeling (Khalifani et al., 2023). In another study, we found head diameter to be the most critical trait influencing performance under normal and saline stress, utilizing RBF, MLP, and CNN models (Khalifani et al., 2022). Therefore, the existing knowledge reinforces the understanding of sunflower adaptive mechanisms and yield determinants.

The consistency among studies and the ongoing identification of these traits highlight their significance in breeding programs to enhance salt tolerance. This emphasizes that these traits can serve as reliable indicators for selecting resilient sunflower lines. Additionally, the integration of machine learning techniques in this study reflects the growing trend in agricultural research towards utilizing high-capacity data for predictive modeling, as reported in recent literature. This shift towards data-driven decision-making encourages the adoption of innovative practices in agriculture, ultimately enhancing research outputs (Leukel et al., 2023; Chlingaryan et al., 2018; Van Klompenburg et al., 2020).

Overall, this study contributes to the existing body of literature on predicting sunflower performance under salt stress by employing advanced modeling methods that demonstrate improved predictive accuracy. The consistency in the identified important traits strengthens the significance of these parameters in assessing sunflower performance under adverse conditions, while highlighting the importance of evolutionary methods for better understanding and managing crop responses to environmental stressors. The results correspond well with previous studies, providing further validation and suggesting that modern modeling approaches can enhance predictive accuracy in the turbulent climate of agricultural production.

Khalifani, S., Darvishzadeh, R., Azad, N., & Rahmani, R. S. (2022). Prediction of sunflower grain yield under normal and salinity stress by RBF, MLP and, CNN models. *Industrial Crops and Products*, *189*, 115762.

Khalifani, S., Darvishzadeh, R., Azad, N., Shayesteh, M. G., Kalbkhani, H., & Akbari, N. (2023). Comparison of influential input variables in the deep learning modeling of sunflower grain yields under normal and drought stress conditions. *Field Crops Research*, *303*, 109145.

Leukel, J., Zimpel, T., & Stumpe, C. (2023). Machine learning technology for early prediction of grain yield at the field scale: A systematic review. *Computers and Electronics in Agriculture*, *207*, 107721.

Chlingaryan, A., Sukkarieh, S., & Whelan, B. (2018). Machine learning approaches for crop yield prediction and nitrogen status estimation in precision agriculture: A review. *Computers and electronics in agriculture*, *151*, 61-69.

**Answer to question 8**

**8. Interpretability of Models: While GEP provides explicit mathematical expressions, how interpretable are the ANN and ANFIS models? Can you provide examples of the rules generated by ANFIS?**

The interpretability of the models varies significantly between ANN, ANFIS, and GEP:

1.	Interpretability of ANN:

ANN is often described as a "black-box" model because it lacks explicit, interpretable relationships between input and output variables. While it provides robust predictions, understanding the specific influence of each input variable on the output is challenging. ANN's interpretability is limited to statistical outputs such as weights or importance scores, which do not provide clear, human-readable explanations.

2.	Interpretability of ANFIS:

ANFIS provides a better balance of predictive power and interpretability. The combination of neural networks and fuzzy logic allows the creation of more comprehensible fuzzy if-then rules. These rules offer an explicit relationship between input variables and the predicted output, which makes ANFIS interpretable.

Example of ANFIS Rules from the Study:

o	Rule 1: If x is A1 (LOW) and y is B1 (HIGH), then f1 = p1x + q1y + r1.

o	Rule 2: If x is A2 (LOW) and y is B2 (MEDIUM), then f2 = p2x + q2y + r2.

Here, A1, B1, p1, q1, and r1 represent the membership functions and coefficients derived during the training phase. The use of subtractive clustering to optimize the number of rules ensures efficient and interpretable model generation.

ANFIS Rules

ANFIS combines fuzzy logic with neural networks, enabling it to create interpretable if-then rules that represent the relationship between input variables and output predictions. In this study, the Sugeno-type fuzzy inference system was used, employing rules like these:

Each rule is defined by:

•	Antecedents (conditions): Membership functions (e.g., LOW, MEDIUM, HIGH) describing the fuzzy sets for each input variable.

•	Consequent (output): A linear equation that relates the input variables to the output, optimized during training.

ANFIS Rule Structure in the Study

For two input parameters such as leaf length (LL) and plant height (PH), the rules can be:

1.	If LL is A1 and PH is B1, then GY = p1 × LL + q1 × PH + r1.

2.	If LL is A2 and PH is B2, then GY = p2 × LL + q2 × PH + r2.

Where:

•	A1, A2, B1, B2 are membership functions.

•	p1, q1, p2, q2 are coefficients learned during training.

•	r1 and r2 are bias terms.

These rules make ANFIS more interpretable than ANN.

•	GEP is the most interpretable model among the three as it provides explicit mathematical expressions representing the relationship between input and output variables. For instance, the equations generated for sunflower grain yield prediction under normal and salinity stress conditions clearly identify the most influential parameters (e.g., LL, HD, HSW, and HDW), providing direct insights into the biological and agronomic processes.

The coefficients in GEP equations indicate the relative importance of variables:

o	Under normal conditions, HSW and HDW have higher positive coefficients, emphasizing their significant contributions to yield.

o	Under salinity stress, LL and HD play critical roles, aligning with their impact on photosynthesis and seed development in stressful environments.

•	The reduction in the number of significant parameters in the salinity stress equation (from six to four) highlights how stress conditions alter the relationships among traits.

3.	Interpretability of GEP:

In general, while ANN is less interpretable, ANFIS improves interpretability through fuzzy rules, and GEP offers the highest interpretability with explicit mathematical formulations.

**Answer to question nine**

**9. Model Performance Metrics: The paper mentions R², RMSE, and MAE. Why were these specific metrics chosen, and how do they complement each other in evaluating model performance?**

**R² (Coefficient of Determination)**

- Measures how much of the variation in grain yield can be explained by the model.
- Values range from 0 to 1, with higher values indicating a better fit.
- Helpful for comparing models and seeing if adding variables or trying different methods improves the model.

**RMSE (Root Mean Square Error)**

- Shows the average size of prediction errors, with extra emphasis on larger errors.
- Sensitive to outliers, making it useful when big mistakes can have serious consequences, like in agriculture.
- Uses the same units as grain yield (e.g., tons per hectare), making it easy to interpret.

**MAE (Mean Absolute Error)**

- Represents the average size of prediction errors straightforwardly.
- Unlike RMSE, it doesn’t emphasize outliers, making it more stable when dealing with extreme values.
- Gives a clear idea of how far predictions are from actual values, on average.

**Complementary Nature of Metrics**

- Using R², RMSE, and MAE together provides a well-rounded evaluation of model performance. Each metric offers a different perspective on the model’s predictive abilities:
- R² indicates how well the model explains the variation in the data.
- RMSE reflects the model's precision by emphasizing larger errors, ensuring that the model is judicious in making predictions.
- MAE provides a straightforward average of errors, giving a clear view of how much predictions deviate from actual values on average.

By considering all three metrics, researchers can identify trade-offs, such as whether a model performs well in explaining variance (high R²) but has some issues with outliers (high RMSE), or alternatively, if it has greater robustness but may not capture the variability as effectively (lower R² with good MAE). Also, these criteria have been used in most grain yield prediction studies, and based on these sources, these criteria were selected to compare the performance of the models.

**Answer to Question ten**

Environmental Conditions: Were there any other environmental factors (e.g., temperature, humidity) monitored during the experiment that could have influenced the results?

During the experimental period, since the trial was conducted under pot conditions, various environmental factors were monitored, including the electrical conductivity levels of the salinity treatments and soil moisture content to ensure adequate irrigation of the plants. While not directly considered environmental factors, nutrient levels were also assessed to ensure that any observed changes in plant performance were primarily due to salinity stress, rather than nutrient deficiencies. Furthermore, the chemical properties of the irrigation water were monitored to prevent unintended stress and maintain precise salinity levels for the plants.

**Answer to Question eleven**

**11. Generalizability of Results: How generalizable are the findings to other sunflower genotypes or different environmental conditions?**

While the findings of this study offer important insights into sunflower yield prediction under salinity stress, their generalizability to other sunflower genotypes and different environmental conditions is constrained by genetic specificity, location-related variability, and the nature of controlled experiments. For broader applicability, it is recommended that further studies test diverse sunflower genotypes across various environmental conditions, thus validating and refining the predictive models developed within this research. These steps will contribute to ensuring that the findings can effectively inform agricultural practices and breeding strategies in varying contexts.

**Answer to question 12**

12. Practical Applications: Can you discuss the practical implications of using these models for farmers and breeders? How can they integrate these tools into their decision-making processes?

These models can significantly enhance decision-making and operational efficiency in various ways. Farmers can make informed decisions regarding planting schedules, resource allocation (such as water, fertilizers, and pesticides), and financial planning by predicting yields based on environmental conditions and key traits. Understanding the impact of salinity enables farmers to optimize irrigation and fertilization practices, thereby minimizing waste and reducing costs. Breeders can identify key traits and develop stress-resistant varieties, accelerating genetic improvement programs. Moreover, these models provide a means to assess the performance of new genotypes under projected climatic conditions, ensuring sustainable sunflower production. Integrating these models with precision agriculture tools, such as remote sensing and soil moisture sensors, can enhance efficiency by providing real-time data on crop performance and environmental conditions. Farmers can tailor management strategies to specific fields based on model outputs. User-friendly tools, including online platforms and mobile applications, empower farmers to obtain accurate predictions based on local data. Training and workshops can facilitate the adoption of these technologies. By predicting adverse conditions, farmers can take proactive measures. Accurate yield predictions can improve financial management and insurance interactions. Ultimately, these models contribute to smart and sustainable agriculture, enhancing resource efficiency.

**Answer to Question thirteen**

13. Limitations and Future Work: What are the main limitations of this study, and how could future research address them?

The study's focus on 96 inbred lines from two specific parents may limit the generalization of findings to a broader genetic base. A more diverse panel of genotypes, including commercial hybrids and wild relatives, should be evaluated across a wider range of genetic traits and responses to environmental stresses to enhance model generalizability. Additionally, pot experiments may not fully capture the complexities of field conditions. Field experiments are recommended to assess model performance under real-world environmental conditions and varying climatic conditions. Furthermore, the study solely investigated salinity stress. The interaction of multiple abiotic stresses, such as drought or heat, should be explored for a more comprehensive understanding and accurate modeling. The study was conducted at a single location; thus, future research should focus on validating models across multiple locations and years to evaluate their predictive power under varying conditions. Finally, the models developed in this study do not leverage advanced technologies such as remote sensing and machine learning to collect real-time data for yield prediction. Future studies should combine these models with precision agriculture technologies, leveraging sensor data, drones, and satellites for ongoing environmental monitoring. This could lead to the development of intelligent decision support systems that continuously refine yield predictions based on ongoing field data.

**Answer to question fourteen**

**14. Statistical Significance: Were the differences in model performance statistically significant? If so, what statistical tests were used to determine this?**

In our study, the performance of ANN, ANFIS, and GEP models was evaluated based on statistical metrics such as the coefficient of determination (R²), root mean squared error (RMSE), and mean absolute error (MAE). Yes, the differences in model performance between ANN, ANFIS, and GEP were statistically significant, as evidenced by the performance metrics (R², RMSE, MAE).

**Answer to Question fifteen**

15. Graphical Representations: The paper includes scatter diagrams and Taylor diagrams. Can you provide more interpretation of these figures and how they support the findings?

The scatter plots illustrate the results obtained from three different models (GEP, ANFIS, and ANN) for predicting a variable (likely crop yield) under two different conditions (normal and saline). Each point in these plots represents an observation, with the expected value from the model plotted on the vertical axis and the observed (actual) value on the horizontal axis. The black diagonal line that runs through the data points is called the regression line, which indicates the best linear fit between the predicted and observed values. The coefficient of determination (R²) reflects the accuracy of the model's predictions; the closer R² is to 1, the greater the model’s predictive accuracy.

In the normal condition (4a), the regression line is represented by the equation (y = 0.496x + 5.853), indicating a linear relationship between the predicted values (y-axis) and the observed values (x-axis). The R² value is 0.512, suggesting that the model can explain approximately 51.2% of the variability in the observed values. In other words, the model accounts for about half of the changes, indicating a moderate correlation between the predicted and observed values.

The scattered data points indicate that the model's predictions vary in accuracy across different instances. In contrast, under saline stress conditions (4b), the coefficient of determination decreases to (R² = 0.402), which is lower than that of the normal condition. This reduction indicates that the model's accuracy diminishes in saline conditions, exhibiting a decreased ability to predict changes in the observed values. Additionally, the increased scatter of the data points in this plot reflects the model's lower accuracy in saline conditions.

For the ANFIS model in normal conditions (5a), the regression line is defined by the equation (y = 0.489x + 6.154), which also indicates a linear relationship between the predicted and observed values. The R² value is 0.574, demonstrating that this model can predict approximately 57.4% of the variability in the observed values, signifying a correlation that ranges from moderate to good. Similar to the previous models, the data points are somewhat scattered, reflecting that the model achieves more accurate predictions in certain instances compared to others. However, in saline stress conditions (5b), the R² value decreases to 0.446, indicating reduced accuracy and greater scatter among the data points.

In the normal condition (6a), the regression line for the GEP model is represented by the equation (y = 0.561x + 5.940\), indicating a linear relationship between the predicted values generated by the model. The R² value is 0.644, which can be explained by the model of approximately 64.4% of the variability in the observed values. In other words, the model has accounted for roughly 64.4% of the changes, indicating a strong correlation between the predicted and observed values. Additionally, the data points are less scattered than in previous models (ANN and ANFIS), indicating the model's higher accuracy under normal conditions. However, under salt stress conditions (6b), the coefficient of determination (R²) decreases to 0.551. This decline indicates that the model's accuracy is reduced in salt stress conditions compared to normal conditions, showing diminished capability in predicting changes in the observed values. Furthermore, the scatter of the data points in this plot is greater than that in the normal condition plot (6a), reflecting the model's lower predictive performance under salinity stress.

Taylor diagrams are powerful tools for comparing the performance of multiple predictive models. These diagrams simultaneously illustrate three important characteristics of model performance:

1. Standard Deviation: This indicates the spread of the predicted values around the mean and reflects the degree of confidence in the predictions.

2. Correlation: This shows the degree of association between predicted and observed values. A high correlation indicates that the model has effectively captured the variations in the observed data.

3. Distance from the Origin: This represents the mean absolute error of the model, indicating how close the predicted values are to the observed values.

Comparison of Model Performance:

In the GEP model, across both conditions, the model is closer to the origin, indicating lower absolute error and better performance than the other two models. Additionally, the angle of this model relative to the horizontal axis is greater, reflecting a higher correlation between the predicted and observed values. The performance of the ANFIS model is generally good; however, it demonstrates slightly weaker performance compared to the GEP model. In both conditions, the ANN model shows the weakest performance, exhibiting inferior results in terms of both absolute error and correlation relative to the other two models.

**Answer to question sixteen.**

**16. How could you clarify the results of ANN models? I mean you should use some cross validation methods such as K-Fold.**

The answer to this question is also fully discussed in the answer to the fifth question.
